# Impact of Provider Health Information Exchange Services on Patient Utilization of Emergency Room and Inpatient Practices in Colorado

**DOI:** 10.3390/jcm14217783

**Published:** 2025-11-02

**Authors:** Darcy Holladay Ford, Rachel Jardim, Kimi Landry, Megha Jha, Isaac Nwi-Mozu, Kelly Joines, Matthew Isiogu

**Affiliations:** 1 Center for Improving Value in Health Care, Denver, CO 80246, USAmjha@civhc.org (M.J.); inwimozu@civhc.org (I.N.-M.); 2 CONTEXTURE, Denver, CO 80222, USAmatthew.isiogu@gmail.com (M.I.)

**Keywords:** health information exchange, all-payer claims database, emergency room visit, inpatient admissions, Colorado, health service research, return on investment, health outcomes, Medicaid

## Abstract

**Background/Objectives:** We wished to determine whether patients of providers subscribing to Health Information Exchange (HIE) services experience a reduction in emergency room visits and inpatient admissions compared to those who do not subscribe and whether the type of HIE service (online access to records via an HIE portal and/or integration with their electronic health record (EHR) and push notifications) a provider uses further impacts utilization. **Methods:** The research team utilized the Colorado All-Payer Claims Database (CO APCD) from 2017 to 2019, intentionally excluding the impact of COVID-19 on HIE services. A pre–post analysis was conducted 15 months before and after the provider subscribed to HIE solutions to quantify the effects when compared to control clinics. **Results:** Subscription to HIE solutions was associated with lower inpatient (IP) admissions than those in a control group for all insurance payer types (*p* = 0.001). The reduction in Medicaid patients was more pronounced when their providers subscribed to either service alone or in combination and was associated with significantly fewer ED and IP admissions than those for control clinics (*p* = 0.003, *p* < 0.05 for HIE portal alone and *p* < 0.0001, *p* = 0.002 for both HIE portal and integrated EHR solutions). HIEs enable providers to coordinate care and access patients’ previous test results and clinical histories, potentially reducing the need for high-cost emergency department and inpatient services. This, in turn, appears to substantially impact low-resource individuals, who have historically been the highest users of these services. **Conclusions:** HIE provider subscription is associated with a reduction in patients’ use of inpatient care after enrolling in HIE products across all insurance types. Medicaid patients experienced the most significant decrease in both emergency room (ED) visits and inpatient admissions when compared to control clinics.

## 1. Introduction

Healthcare costs continue to rise at an alarming rate, increasing from 2022 to 2023 by over 7.5% to USD 14,570 per capita and USD 4.9 trillion [1]. The facilitated exchange of patient health information between healthcare providers is a crucial step toward a more efficient and effective healthcare system, which can help reduce ever-increasing costs [2,3]. The 2009 Health Information Technology for Economic and Clinical Health (HITECH) Act provided financial incentives to providers to adopt electronic health records (EHRs). It supported the development of HIEs to promote data sharing and care coordination [4]. Positive subscriber feedback and increased demand for HIE suggest that providers highly value how it reduces administrative burden and increases administrative efficiency by an estimated 120 min per patient per year [5,6,7,8].

HIE’s direct impact on healthcare claims has not been studied to date. Previous HIE studies that have estimated impacts, cited through survey studies, indicate that HIE services enable providers to quickly access patients’ complete medical records before and during visits, thereby allowing potential ED visits, rehospitalizations, and mortality to be reduced and improving access to outpatient care [9,10]. When HIEs send push notifications about patient needs directly to providers, providers report that it reduces inpatient admissions, length of stay, and emergency department visits [6]. Additionally, HIE services are reported to help reduce duplicate care, including imaging, laboratory work, and visits for obtaining medical records, resulting in a 44% to 67% reduction in repeat or redundant emergency department imaging [11,12,13].

Previous HIE survey studies indicate that services also save money for patients and the healthcare system. For example, HIE access in emergency departments has an estimated cost savings of up to USD 2000 per patient by preventing redundant laboratory tests, imaging, consultations, and hospital admissions [5]. These benefits are particularly impactful for under-resourced communities, including those enrolled in Medicare and Medicaid, who utilize emergency department care at a higher rate and have fewer resources for preventive services than better-resourced populations [14,15].

While HIE services are frequently cited as saving time and money and improving outcomes, few studies have rigorously quantified their impact. Existing evidence primarily comes from small observational studies [9,15,16,17]. This study utilized the Colorado All-Payer Claims Database (CO APCD), a comprehensive database of dental, vision, medical, and pharmacy claims from 2009 to 2023 for all payers in the state, as well as for 2024 and 2025 for commercial and Medicaid payers. This allowed the research team to compare providers who subscribed to HIE services with a control group and measure actual emergency room and inpatient utilization across all settings. The results were further stratified by insurance payer type, including Medicaid, commercial, and Medicare plans.

Some HIE solutions can be administratively burdensome, as providers may be required to log in and out frequently due to administrative tasks and to access patient records [8,13,18,19]. This analysis compared two types of HIE services aimed at reducing this burden: an HIE web-based portal service requiring provider login and a patient-profile-integrated/EHR service, including push notifications and auto-login, to understand the impact of HIE services on patient health utilization. The physician group purchases these services for the entire clinic, allowing all providers to access patient clinical information from care received in outside settings.

This study aimed to determine whether patients of providers who subscribe to Health Information Exchanges (HIE) solutions, including online access to records via an HIE portal and integration with their electronic health record (EHR), experience a reduction in emergency department (ED) visits and inpatient admissions compared to those who do not subscribe to these solutions.

The following research questions guided this study:

(1) What is the impact of providers subscribing to HIE services on patient emergency room visit rates and inpatient admissions compared to a control?
(2) Does the type of provider-adopted HIE service influence the effects of HIE subscription on patient utilization?
(3) Does the patient insurance payer type influence the impact of HIE subscription on patient utilization of emergency rooms and inpatient facilities?

## 2. Materials and Methods

This study was completed using CO APCD data, a robust dataset comprising over 1 billion medical, dental, and pharmaceutical claims from approximately 75% of residents specifically of Colorado who have health insurance in the state, which included 5.5 million individuals from 2009 to 2025. The information contained in the CO APCD includes claims from Commercial, Medicare fee-for-service, Medicare Advantage, Medicaid, and voluntarily submitted self-pay plans.

The solutions offered through Colorado’s statewide HIE include two information delivery services: (a) the HIE portal, a web-based portal where providers can look up a patient’s complete medical record, and (b) the integrated EHR solution, a customizable service that integrates with a provider’s electronic medical record system to update patient records with new test results, imaging, provider notes, and patient information, allowing for the patient’s information and outside medical information to seamlessly integrate into the EHR without the provider having to log into a web based portal. All providers who subscribe to (b) also gain access to (a).

The research team implemented a control pre–post design to analyze the impact of HIE enrollment on patient emergency room and inpatient utilization. The research team received a list of clinics that had subscribed, allowing all providers in the clinics to access the HIE solution from the primary Colorado HIE services between 2017 and 2019, specifying the type of their subscription (portal or EHR/portal). The 2017–2019 period was selected to examine the impact of provider subscription on healthcare utilization in HIE services prior to the COVID-19 pandemic, to understand the effects of HIE enrollment without the COVID-19 pandemic’s impact on care utilization Additionally, the clinics selected were chosen based on their continued enrollment for over three years in HIE services and the availability of Medicare fee-for-service data in the Colorado All-Payer Database, which is typically delayed by over two years. The chosen control clinics were those that had subscribed to HIE products after 2020 but not before and were ancillary clinics similar in terms of the population served and the number of patients served per clinic. The control group’s “intervention date” was 1 January 2018.

The research team compared all patients’ medical healthcare claims data from 15 months preceding and following enrollment in HIE services for each clinic between 2017 and 2019, as each clinic onboarded to the new HIE solutions at different times during the period, to assess the impact of provider HIE subscription on emergency room visits and hospitalizations in the subscribing and control clinics. The researchers used the Provider NPI, Billing Provider NPI, and service location zip code to isolate these claims for each patient seen in either the intervention or control clinics. Each patient’s claims were assigned to one of the clinics in the cohort and a specific product type (integrated EHR and HIE portal, portal alone, or control). To capture only healthcare users and improve the comparison to HIE-subscribing clinics, the research team included only individuals in the CO APCD who had at least one medical healthcare claim of any type (outpatient or inpatient) during the study period in this cohort. The research team calculated the per-member-per-year rates for all study measures by summing the number of member with insurance coverage every month, allowing for an adjustment for members with fewer than 12 months of eligibility during the study years.

The researchers utilized DBeaver SQL (DBeaver Corp., Wilmington, DE, USA) and SAS software, version 9.4 (SAS Institute Inc., Cary. NC, USA), for all analyses. They calculated the aggregate primary outcomes by product type (control, integrated EHR + HIE portal, and HIE portal only) and insurance payer type (Medicaid, Commercial, Medicare fee-for-service, or Medicare Advantage). The variables were tested for normality and to identify outliers. The clinics were compared pre to post for each product through descriptive statistics, and then, the change from pre to post across products was examined through type, conducting comparisons independently against the control clinic. The two-tailed *t*-test was selected because only two groups were being compared at any given time, and it offers slightly more power than ANOVA in two-group comparisons due to the fewer degrees of freedom used.

## 3. Results

The final cohort across the three groups, including the HIE portal, HIE plus EHR, and the control, consisted of 42,213 unique members, spanning both pre- and post periods. Additionally, the cohorts had 557,252 unique emergency room visits and inpatient care claims. Finally, the number of members with health insurance coverage increased from the pre to the post periods across all product types (Table 1).

### 3.1. Emergency Department Admissions

**Figure 1:**
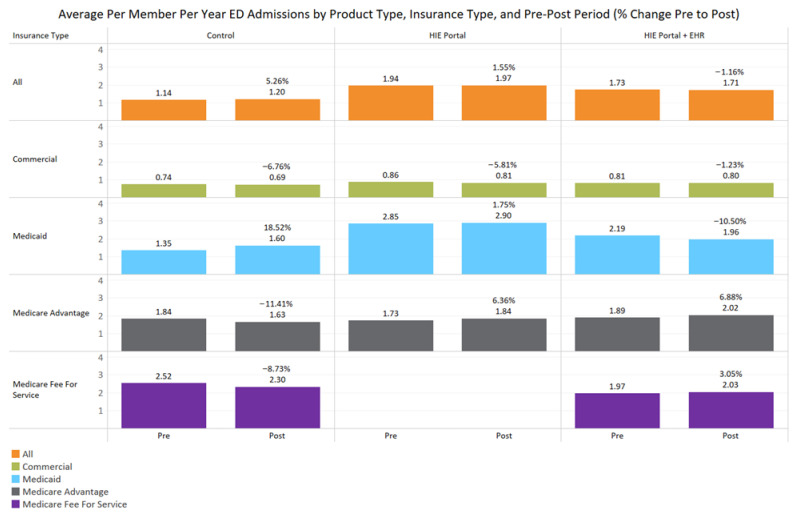
This figure depicts the percentage change in emergency room utilization between the pre and post periods for control, HIE portal products, and the integrated HIE EHR plus portal product. No Medicare fee-for-service claims were observed for the patients of HIE-subscribing providers. The primary insurance payer of the pre–post period was used to identify each member uniquely for analysis.

Medicaid patients experienced an 11% decrease in emergency room visits from pre- to post-intervention when the provider used integrated EHR + HIE portal services, compared to an 18.5% increase in the control group (*p* < 0.00001, Table 2, Figure 1); the HIE portal alone also experienced a relatively lower increase in ED admissions (2.8% compared to Medicaid enrollees in the control group, *p* = 0.003, Table 3, Figure 1). HIE solutions resulted in a relative reduction in emergency room visits across all the combined insurance payer group compared to those in control clinics, despite minor increases observed between HIE products and controls for commercial and Medicare fee-for-service; however, this decline was not statistically significant.

### 3.2. Inpatient Admissions

**Figure 2:**
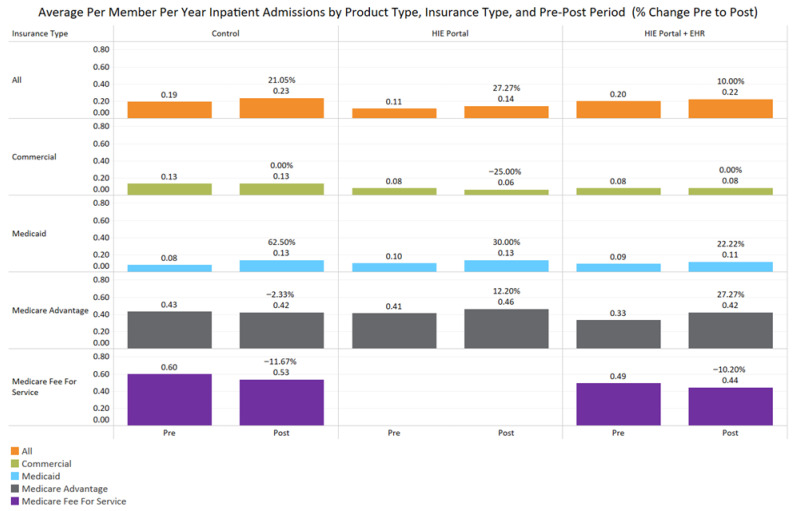
This figure depicts the change in inpatient admission utilization between the pre and post periods for control, HIE portal products, and the integrated HIE EHR plus portal product. No Medicare fee-for-service claims were observed for the patients of HIE-subscribing providers.

Patients post-intervention: Control clinics experienced an average increase of 21.1% in per-member-per-year (PMPY) inpatient admissions from pre-intervention to post-intervention for all insurance payer types (Figure 2). Patients of providers subscribing to integrated EHR HIE and portal services, as well as those using HIE portal services alone, also experienced an average increase in per-member-per-year inpatient admissions over time, although they were not significantly different from each other. However, the HIE-integrated EHR plus portal services group had a significantly lower increase in admissions from pre- to post-treatment (10%) compared to that for patients in the control group (*p* = 0.001, Table 2, Figure 2). This finding was replicated in Medicaid enrollees, with a 62.5% increase in per-member-per-year (PMPY) inpatient admissions from pre- to post for the control group (Table 3). In comparison, the increases for both HIE product groups were significantly lower among Medicaid patients, for enrollees using an HIE portal alone (30%, *p* = 0.04) and those using integrated EHR HIE plus portal services (22.2%, *p* = 0.001; Table 3, Figure 2).

## 4. Discussion

Increasing the number of provider subscribers signifies high value in HIE solutions; however, there is a lack of quantifiable information on the value of these products [5,6,7,8]. This analysis differed from other similar studies in that the post period included any emergency room visit or hospitalization, not just readmissions, and was not limited to ambulatory-sensitive conditions, thereby increasing the refinement of the conclusions [11,12,13,20,21,22,23,24,25,26,27,28,29,30]. The current research study evaluated the impact of providers subscribing to HIE products on patient utilization of emergency department visits and inpatient admissions, observing a decrease in inpatient admissions for HIE portal subscriptions and EHR integration, with a greater reduction observed for EHR-integrated services. This finding aligns with previous observational survey research studies, which indicate that providers who utilize EHRs through HIE services reduce the rate of inpatient admissions, particularly for specialty providers who require more coordination with primary care providers [20]. The emergency room reduction for HIE products across all insurance payer types was not significantly significant. Still, it outperformed the control clinics with an increase of 5.3%, compared to the HIE portal alone (2.6%) and the integrated EHR and web portal (1.8%).

Compared to the control group, the integrated EHR and HIE portal resulted in a reduction in emergency room visits and inpatient admissions among Medicaid enrollees, suggesting potential protection for this vulnerable population. The differentially positive impacts of HIE subscription on the Medicaid population may suggest an easier ability for these providers to access patient records with a lower administrative burden, allowing providers to support their patients and reduce their rate of potentially avoidable emergency room visits [11,12,13,20,21,22,23,24,25].

Compared to the HIE portal, the integrated EHR solution plus portal resulted in a reduction in emergency room visits and inpatient hospitalizations. The ability of providers to access information without needing to use a separate portal while still being able to query if needed likely suggests that providers found it easier to integrate it into their clinical practice, thereby reducing the administrative burden for providers to coordinate efficient care for their patients [5,6,7,8,20,21,22,23]. There has been an advancement in the atmosphere of the federal Trusted Exchange Framework and Common Agreement (TEFCA), allowing for the facilitated exchange of health and human services data across states [20,21,22,23,24,25,26,27,28,29,30]. However, a common criticism of these services is that they require providers to log in and query each patient, which may reduce the likelihood that a provider will look up patients, potentially leading to misjudgment and underestimating the complexity of their patients’ clinical picture, thereby lowering the benefits of HIE services [22,23,24,25,26,27,28,29,30]. Although a 2023 New York Medicare fee-for-service analysis reported an increased odds of reducing emergency and inpatient admissions with query-based services, the availability of both EHR integration and query-based HIE services, as demonstrated in this study, may still enable providers to navigate care for their patients of all ages and insurance payer types [31].

This study has financial implications for providers, payers, and HIE systems. Providers are motivated to deliver quality healthcare to their patients through value-based care, which includes incentivizing the coordination of care when patients transition from emergency or hospital systems [32]. The integration of quality and resource use measures, often built into this reimbursement structure, aligns well with the value of HIE systems, including the increased ability to have a broader view of a provider’s patients’ care delivered in other non-provider-affiliated locations. The costs of inpatient admissions and emergency room visits contribute to the growth in healthcare costs, which are often targeted by payers as a source of reduced costs when they can be reduced.

The Rural Health Transformation Program (RHTP), established under H.R. 1, provides USD 50 billion in federal grants to help states modernize rural healthcare [33]. CMS’s first funding opportunity, released September 15, confirms that health IT, including HIE services, software, hardware, and provider training, is an eligible expense.

This aligns directly with our findings: Medicaid patients in our study experienced statistically significant reductions in emergency and inpatient utilization, measured through actual claims. States now have a clear opportunity to invest in HIE infrastructure that lowers costs and improves outcomes.

For rural and safety net providers—such as clinics, critical access hospitals, and behavioral health centers—RHTP funding enables long-overdue connectivity. HIE supports real-time care coordination and provider notifications, turning data exchange into continuity of care [34].

### 4.1. Limitations

Pre–post evaluations are designed to provide a snapshot of the changes between the two time periods of interest. Additionally, pre and post evaluations are constrained by the maturation effect; changes over time may reflect an aging population or other global factors influencing utilization, rather than changes resulting from the intervention.

The CO APCD does not capture individuals insured by TRICARE, 50% of self-insured ERISA plans, or uninsured Colorado residents; therefore, this analysis is limited in its ability to estimate the impact of HIE utilization on these populations. The 2020 SARS-CoV-2 pandemic was intentionally avoided; however, unmeasured environmental and historical factors may have impacted outcomes. Additionally, to mitigate the impact of the 2020 SARS-CoV-2 pandemic and due to the limited Medicare fee-for-service during the study period, this analysis included data from 2017 to 2019. Additional analysis is recommended using more up-to-date data to understand the implications of the post-COVID-19 healthcare landscape better.

The level of HIE utilization by the providers was not verified in this study; instead, the clinics had continuous access throughout the study period. The integrated HIE solution seamlessly integrates into a patient’s EHR, making uptake easier for the provider.

### 4.2. Future Research

Although 15 months before the intervention is a significant period for establishing a baseline for clinics, more time is likely needed to fully assess the impact of HIE products on changing healthcare behaviors. To better understand the differential effects of HIE provider enrollment on patient outcomes and expenditures, additional research should consider extending the patient observation period beyond 15 months of provider subscription and adjusting for patient demographic information, such as acuity, comorbidities, age, socioeconomic status, and clinic attended. Additional research should assess whether there are differences between clinics in adapting HIE products into their workflow, with greater time likely reducing variation.

## 5. Conclusions

United States healthcare costs are increasing at an alarming and unsustainable rate. HIE products that providers use to coordinate care and review previous patients’ test results and clinical histories appear to decrease high-cost emergency department and inpatient services, substantially impacting low-resource Medicaid individuals, who have historically been the highest users of these services. These findings suggest that patients may also experience reduced inpatient admissions and improved outcomes after their providers enroll in integrated EHR and HIE portal products.

## Figures and Tables

**Table 1:** Cohort demographics.

	Control		HIE Portal		HIE Portal + EHR	
	*Pre* (*N* = 11,337)	*Post* (*N* = 14,236)	*Pre* (*N* = 7524)	*Post* (*N* = 8071)	*Pre* (*N* = 11,738)	*Post* (*N* = 12,813)
**Insurance Type**						
Commercial	5693	6560	3200	3246	4750	5144
Medicaid	1620	1718	3022	3147	4431	4803
Medicare	3408	3714	1165	1233	1657	1684
Medicare Advantage	1220	2979	377	697	1379	1664
**Age Groups**						
Less than 18 years	2378	2672	868	1198	4011	4062
18–40	1789	2188	2617	2616	2203	2720
41–64	2878	3175	2715	2568	2726	2983
65 and above	4378	6346	1389	1774	2915	3199
**Race and Ethnicity**						
AIAN	15	15	24	20	16	18
Asian	555	713	53	70	125	137
Black	100	128	342	355	489	536
Hispanic or Latino	291	423	327	328	1747	1720
Native Hawaiian or other Pacific Islander	632	716	604	616	682	756
Unknown	3945	4611	2459	2609	4502	4886
White	5799	7630	3715	4073	4177	4760
**Gender**						
Female	6604	8331	4761	5106	6330	7036
Male	4707	5891	2758	2961	5391	5772
**County Type**						
Null	110	130	198	194	179	197
Rural	167	194	146	147	124	216
Urban	11,060	13,912	7180	7730	11,435	12,400

Footnote: Individuals are not mutually exclusive and may be counted in more than one category.

**Table 2:** *t*-test comparisons for all insurance payer types.

Utilization	Control	% Change	HIE Portal	% Change	EHR + HIE Portal	% Change
PMPY ED Visits	0.06	5.3%	0.05	2.6% *p* = 0.727	0.03	1.8% *p* = 0.161
PMPY Inpatient Admissions	0.04	21.1%	0.03	27.3% *p* = 0.771	0.02	10.0% *p* = 0.001 *

* Statistically significant at *p* < 0.05.

**Table 3:** *t*-Test Medicaid HIE Services vs. Control Comparisons for Utilization.

Utilization	Control	% Change	HIE Portal	% Change	EHR + HIE Portal	% Change
PMPY ED Visits	0.25	18.5%	0.08	2.8% *p* = 0.003 *	−0.11	−5.3% *p* < 0.00001 *
PMPY Inpatient Admissions	0.05	62.5%	0.03	30.0% *p* = 0.047 *	0.02	22.2% *p* = 0.002 *

* Statistically significant at *p* < 0.05.

## Data Availability

The data supporting these findings are available within the article or upon request.
